# P22 Small Noncoding RNAs Are Actively Secreted in *Salmonella* Outer Membrane Vesicles During Bacteriophage Infection

**DOI:** 10.3390/ncrna12040021

**Published:** 2026-06-26

**Authors:** Sayema Naaz, Haley A. Kominek, Lydia A. Hayes-Guastella, Autumn M. McDaniel, Enas S. Alsatari, Adeyeye I. Haastrup, Olivia G. Clark, Devin M. Katerski, Francois O. Prinsloo, Olivia R. Roberts, Meredith A. Shaddix, Bridgette N. Sullivan, Isabella M. Swan, Emily M. Hartsell, Jeffrey D. DeMeis, Suhas S. Patil, Richard H. Pham, Makala R. Cox, Glen M. Borchert

**Affiliations:** 1 Department of Pharmacology, University of South Alabama, Mobile, AL 36608, USA; sn2227@jagmail.southalabama.edu (S.N.); had2221@jagmail.southalabama.edu (H.A.K.); aih2121@jagmail.southalabama.edu (A.I.H.); emh2124@jagmail.southalabama.edu (E.M.H.); ssp2221@jagmail.southalabama.edu (S.S.P.); rhp2221@jagmail.southalabama.edu (R.H.P.); mc2328@jagmail.southalabama.edu (M.R.C.); 2 Stokes School of Marine and Environmental Sciences, University of South Alabama, Mobile, AL 36688, USA; lah2222@jagmail.southalabama.edu; 3 Department of Biomedical Sciences, University of South Alabama, Mobile, AL 36688, USA; amm1821@jagmail.southalabama.edu (A.M.M.); mas1929@jagmail.southalabama.edu (M.A.S.); 4 Department of Pathology, University of South Alabama, Mobile, AL 36617, USA; esa2222@jagmail.southalabama.edu; 5 Department of Biology, University of South Alabama, Mobile, AL 36608, USA; ogc1921@jagmail.southalabama.edu (O.G.C.); dmk2122@jagmail.southalabama.edu (D.M.K.); fop1921@jagmail.southalabama.edu (F.O.P.); orr1921@jagmail.southalabama.edu (O.R.R.); bns1226@jagmail.southalabama.edu (B.N.S.); ims2022@jagmail.southalabama.edu (I.M.S.); 6 Department of Molecular Biology, Massachusetts General Hospital, Boston, MA 02114, USA; demeis@molbio.mgh.harvard.edu

**Keywords:** P22, *Salmonella*, outer membrane vesicle, noncoding RNA, bacteriophage, sRNA

## Abstract

**Background/Objectives**: Outer membrane vesicles (OMVs) are membrane-encapsulated spherical structures ~120 nm in diameter derived from Gram-negative bacterial cell envelopes. OMVs are primarily generated by outer membrane blebbing but contain proteins, DNA, and RNAs at concentrations distinct from that of the intracellular complement. OMVs have been associated with a number of different cellular functions including intercellular communication and resistance to phage. **Methods**: As bacterial small RNAs (sRNAs) also participate in bacteriophage defense and are specifically delivered to and enriched in OMVs, we recently elected to examine the effects of P22 infection on Salmonella cytosolic and OMV sRNA abundance by employing RNA sequencing. **Results**: We find that P22 infection triggers a global reduction in sRNAs (with Salmonella sRNA expression levels averaging only 15.6% those observed in noninfected cells) coupled with a reciprocal 72.7% global increase in Salmonella tRNA expression levels. Additionally, of note, while OMV small noncoding RNA (sncRNA) abundance is normally ~1/10 that found in the cytosol, we find that P22 infection triggers active OMV encapsulation and secretion of: (1) a subset of sRNAs, (2) all Salmonella tRNAs including one highly complementary to the P22 genome, and, much to our surprise, (3) ten distinct sRNAs expressed from P22. **Conclusions**: In summary, the work presented here identifies several Salmonella sncRNA cytosolic and/or OMV abundances significantly altered during P22 infection, and to our knowledge, this constitutes the first reported characterization of bacteriophage-encoded sRNAs being actively secreted within host OMVs.

## 1. Introduction

The P22 bacteriophage, a member of the Caudoviricetes class and genus of Lederbergvirus, specifically targets *Salmonella* Typhimurium (a Gram-negative, rod-shaped, non-spore-forming, facultative anaerobe belonging to the Enterobacteriaceae family) [1]. Sharing genetic and regulatory similarities with bacteriophage λ, P22 is classified as a temperate double-stranded lambdoid DNA phage [2]. Based on the multiplicity of infection (MOI), P22 can follow either a lytic or lysogenic growth pathway. The lytic pathway leads to immediate viral replication and cell lysis, while the lysogenic pathway involves the integration of the phage chromosome into the host genome. The versatility of P22 makes it an indispensable asset in the exploration of bacterial genetics and the gold standard for bacteriophage studies of *Salmonella* Typhimurium [3,4]. Additionally, understanding phage gene expression is crucial for comprehending how phages regulate their development within bacterial cells and for gaining insights into the genetic information they transfer. This is particularly important for phages that transfer key virulence genes, which can convert non-pathogenic bacterial hosts into virulent strains or enhance the virulence of pathogenic bacteria by providing them with more effective mechanisms of attachment, invasion, or evasion of the host immune defense [5].

Similarly involved in information transfer, outer membrane vesicle (OMV) release by *Salmonella* is now well documented [6]. Approximately 120 nm in diameter [7], bacterial OMVs are extruded from the cell and largely constitute miniature fragments of the cell surface. Containing an array of macromolecules, these bilayered, membranous structures facilitate communication by delivering a range of biomolecules—including proteins, lipids, and nucleic acids—to other bacteria and/or host cells [8,9,10,11]. Of note, *Salmonella* OMVs can significantly inhibit P22 bacteriophage infection, and these OMV-driven bacteriophage inhibitions are largely thought to occur through OMVs acting as decoys, binding to and neutralizing phages [12]. Because OMVs contain the same surface receptors as the cell membrane, phages conceivably bind to the vesicles instead of the bacteria, effectively reducing the efficacy of phage infection.

Likewise relevant to the work presented herein, bacterial small RNAs (sRNAs) also participate in bacteriophage defense. As an example, *Vibrio cholerae*-encoded sRNAs directly antagonize the replication of the lysogenic phage VP882 by downregulating phage mRNA expression levels. Interestingly, to counteract this during activation, VP882 expresses its own Hfq-binding sRNAs in order to outcompete host-encoded sRNAs. At least one of these VP882-encoded Hfq-binding sRNAs, VpdS, has been shown to directly promote phage replication by regulating both host and phage mRNAs [13].

As (1) *Salmonella* OMVs significantly inhibit P22 bacteriophage infection [12], (2) some bacterial sRNAs also directly participate in bacteriophage defense [13], (3) OMVs can be taken up by neighboring cells allowing for the delivery of encapsulated RNAs [8,9,10,11], and (4) bacterial sRNAs are specifically delivered to and enriched in OMVs via Hfq [14], we recently elected to determine the effects of P22 infection on *Salmonella* cytosolic and OMV sRNA abundance. To examine this, we infected *Salmonella* with the P22 bacteriophage then sequenced distinct small RNA populations isolated from whole cell lysates and OMVs.

## 2. Results

### 2.1. Small Noncoding RNA (sncRNA) Sequencing

**Figure 1:**
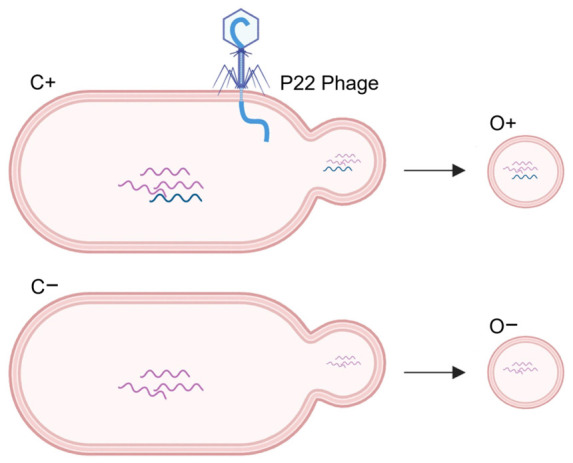
Cartoon of RNA isolates. C+ indicates RNA isolated from *Salmonella* cells infected with P22 bacteriophage; O+ indicates RNA isolated from outer membrane vesicles secreted from *Salmonella* cells infected with P22; C− indicates RNA isolated from *Salmonella* cells not infected with P22; O− indicates RNA isolated from outer membrane vesicles secreted from *Salmonella* cells not infected with P22. Purple wavy lines indicate *Salmonella* RNAs. Blue wavy lines indicate RNAs expressed from P22.

To identify sncRNAs expressed by *Salmonella* P22 during infection, four separate RNA isolates were collected from *Salmonella* enterica serovar Typhimurium (strain LT2) cultures on three separate occasions, and ~20 million high-quality reads were generated from each. As illustrated in Figure 1, C+ RNA refers to cytosolic RNA isolated from *Salmonella* cells infected with the P22 bacteriophage; O+ RNA refers to RNA isolated from OMVs secreted from *Salmonella* infected with P22; C −RNA refers to cellular RNA isolated from *Salmonella* cells not infected with P22; and O− RNA refers to RNA isolated from OMVs secreted from *Salmonella* not infected with P22. In agreement with a recent examination of *E. coli* OMVs finding comparable OMVs averaging 123.9 nm in diameter [7], the ZetaView visualization of our O−OMV isolates identified a single, similarly sized particle pool averaging 122.7 nm in diameter. Notably, in addition to observing a particle pool averaging ~120 nm in O+ OMV isolates, distinct particle pools averaging 70.1 nm and 208.6 were also detected, and based on previous analyses [15], they were presumed to correspond to P22 phage and OMV:P22 associated particles, respectively (Supplemental Scheme S1).

### 2.2. Salmonella sncRNAs Affected by P22

**Figure 2:**
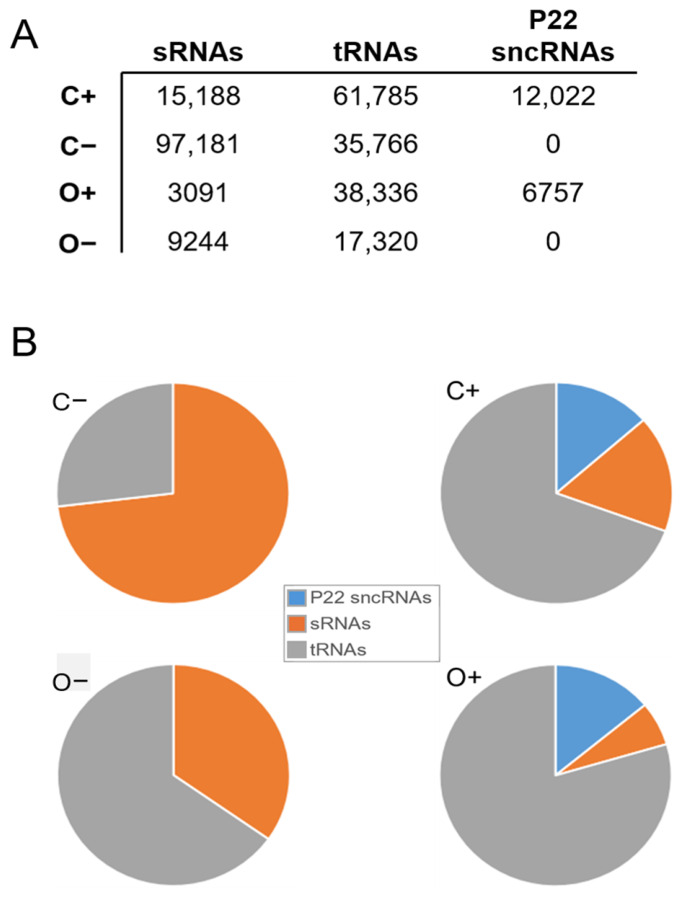
Effects of P22 infection on *Salmonella* sncRNA profiles. (**A**) Number of reads per million aligning to sRNAs (annotated *Salmonella* sRNAs), tRNAs (annotated Salmonella tRNAs), and P22 sncRNAs (ten unique P22 sncRNAs described in this study). C+, C−, O+, and O− refer to isolates as defined in Figure 1’s legend. (**B**) Pie charts depicting percentages of reads aligning to total number of sRNAs, tRNAs, and P22 sncRNA alignments.

All reads were independently aligned to the LT2 genome. Whereas nearly 10% (97,181 reads per million (RPM)) of C− reads corresponded to one of the 929 *Salmonella* sRNAs reported to date, the percentage of reads aligning to annotated sRNAs was reduced to only 1.5% (15,188 RPM) during P22 infection. Reads corresponding to *Salmonella* sRNAs were similarly reduced in OMV isolates during P22 infection, from 9244 RPM in O− to 3091 RPM in O+. The cytosolic expression of only one appreciably expressed (>25 RPM) sRNA, FinP, was increased by ≥100% during P22 infection. Conversely, the cytosolic expression levels of 151 sRNAs appreciably expressed (>25 RPM) in noninfected cells decreased to less than half their normal expression levels during P22 infection (Table 1). In stark contrast to sRNA effects, P22 infection resulted in an approximate doubling of tRNA abundance in both the cytosol (from 35,766 RPM in C− to 61,785 RPM in C+) and OMVs (from 17,320 RPM in O− to 38,336 RPM in O+) (Figure 2, Table 1). Strikingly, the cytosolic expression levels of all tRNAs were increased by at least 50% during P22 infection. Interestingly, however, while most tRNA OMV abundances were also increased during P22 infection, the expression levels of tRNA^Asp^ and tRNA^Met^ were markedly depleted in OMVs during infection (Table 1). Similarly, we found that 37 of 45 sRNAs with appreciable OMV expression levels (>25 RPM) decreased to less than half their normal expression levels during P22 infection. Finally, we observed clear differences between cytosolic and OMV sRNA profiles potentially indicative of the selection and active loading of specific sRNAs. Whereas most sRNAs are significantly more abundant in cytosolic than OMV isolates, we found the OMV abundances of four sRNAs with appreciable OMV expression levels to be roughly doubled during P22 infection (e.g., FinP and SsrS), and furthermore, the OMV expression levels of several sRNAs (e.g., FinP, IsrB2 and npcRNA44) were comparable to cytosolic levels (Table 1).

### 2.3. SncRNAs Expressed by P22

**Figure 3:**
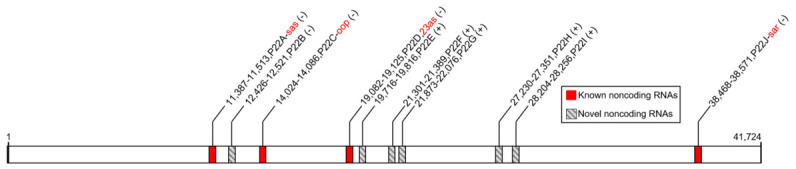
SncRNAs transcribed from P22 genome during phage infection. Scale schematic of P22 genome (1–41,724 basepairs) with specific sncRNAs represented by shaded rectangles. SncRNA names assigned in this study (P22A-J arbitrarily assigned by position), genomic positions, strands (+ or -), and previously reported names are indicated. Previously reported sncRNA positions and names are shown in red.

**Figure 4:**
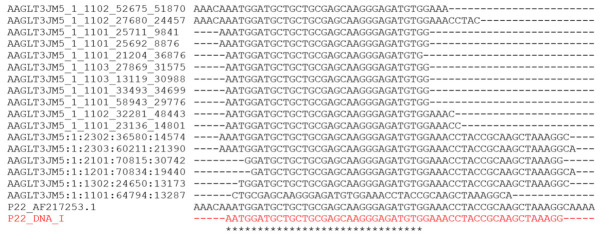
P22I-defining reads. Alignment of P22 genome AF217253.1 (middle), P22I sncRNA (red), and select O+ reads (top) used to define P22I. P22_DNA_I indicates final sncRNA consensus sequence call (as included in Table 1). Final sncRNA calls required each position to be included in ≥80% of full set of aligning reads. “*” signifies nucleotide alignment.

**Figure 5:**
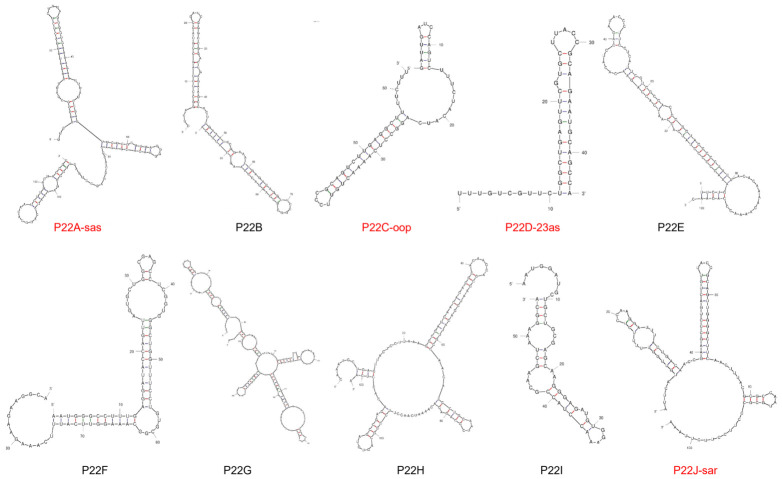
P22 sncRNA secondary structures. The most thermodynamically stable secondary structures predicted by MFold [21]. The names of previously reported sncRNAs are indicated in red.

All reads were also independently aligned to the P22 genome. We found that the majority of reads perfectly aligning to the P22 genome identically aligned to a 46 nt sequence 100% conserved in the *Salmonella* tRNA^thr^ genomic locus. That said, while these reads aligned to both genomes, over 99% of these reads additionally aligned to a flanking sequence specific to the *Salmonella* genome, confirming that they correspond to *Salmonella* transcripts. In addition to these tRNA-aligning reads, large numbers of reads perfectly aligning to the P22 genome, but not the *Salmonella* genome, were also identified, and critically, these reads only occurred in P22-infected isolate datasets. A total of 99.8% of these reads aligned to ten unique positions in P22, accounting for less than 3% of the total genomic sequence. As such, these positions were considered putative P22 sncRNA loci collectively expressing ten sncRNAs appreciably expressed from the P22 genome during infection (Figure 3, Table 2). Importantly, we found that four of these loci correspond to the only four P22 sncRNAs reported to date: *sas* [16], *oop* [17,18], *23as* [19], and *sar* [20]. That said, we found each of the six remaining hitherto unreported putative sncRNAs well-supported by significant numbers of independent reads (see Figure 4 for example P22I alignments) and validated the identities of two of the putative sncRNAs by employing a series of specific internal and flanking primers for RT-PCR (Supplemental Scheme S2). Notably, one of the previously characterized sncRNAs we found to be appreciably expressed from the P22 genome during infection, *sar*, has been described in a number of distinct bacteriophage genomes [21], and as such, we elected to examine whether the novel sncRNAs identified in this work might be similarly conserved. Importantly, we found each of these novel sncRNAs identically maintained in a number of additional bacteriophage genomes (Supplemental Table S1), and furthermore, we found notable similarities between the secondary structures of each of these novel sncRNAs and the characteristic hairpin-heavy structure of *sar* sncRNA [21,22] (Figure 5).

Perhaps most notably, we find P22 sncRNAs present in both the cytosol and OMVs. That said, whereas *Salmonella* sRNAs are slightly more abundant than P22 sncRNAs in the cytosol (15,188 RPM to 12,022 RPM), P22 sncRNA abundance is more than double that of *Salmonella* sRNAs in OMVs (6757 and 3091 RPM, respectively) potentially indicating the preferential loading of P22 sncRNAs into *Salmonella* OMVs (Figure 2).

### 2.4. P22 sncRNA Putative Targets

Each of the four previously reported P22 sRNAs occur antisense to (embedded within and on the opposite strand of) protein-coding genes. In contrast to these antisense sRNAs, the six hitherto uncharacterized P22 sRNAs are each located on the same strand and just upstream (average 21 nt) of a protein-coding gene. As such, all ten P22 sncRNAs can be placed into one of two categories: (1) sncRNAs with 3′ ends located just upstream of a protein-coding gene on the same strand and (2) sncRNAs overlapping and antisense to a protein-coding gene. Six sncRNAs fall into Group 1 as follows: The 3′ end of P22B is 16 nt from the start codon of Gene 24 [2,23]. The 3′ end of P22E is 48 nt from the start codon of Gene 13 [24,25]. The 3′ end of P22F is 6 nt from the start codon of rha. The 3′ end of P22G is 16 nt from the start codon of ORF 25. The 3′ end of P22H is 17 nt from the start codon of Gene 1 (portal protein) [26]. And the 3′ end of P22I is 23 nt from the start codon of Gene 5 (coat/capsid protein) [27]. Interestingly, each of these genes is found sandwiched between two protein-coding genes located on the same strand as the sncRNA. In light of this, these sncRNAs could conceivably function analogously to the sncRNA IsrK encoded by the Gifsy-1 prophage of *Salmonella*. The 3′ end of IsrK RNA is located only 29 nt from the start codon of a downstream ORF, and the production of the upstream toxic AntQ protein is directly regulated by an interaction between IsrK sncRNA and the downstream ORF [28]. The remaining four sncRNAs fall into Group 2, and each of these has been previously reported. These include: P22A-sas, which is antisense to SieB [16]; P22C-oop, which is antisense to C1 [17,18]; P22D-23as, which is antisense to Gene 23 [19]; and P22J-sar, which is antisense to Ant [20] (Table 3).

## 3. Discussion

OMVs have been associated with a number of different cellular functions such as intercellular communication (including delivery of encapsulated RNAs) [8,9,10,11] and resistance to phage (e.g., *Salmonella* OMVs significantly inhibit P22 bacteriophage infection [12]). As bacterial sRNAs also participate in bacteriophage defense [13] and are specifically delivered to and enriched in OMVs via Hfq [14], we recently elected to examine the effects of P22 infection on *Salmonella* cytosolic and OMV sRNA abundance.

We found that P22 infection triggers a global reduction in sRNAs (with *Salmonella* sRNA expression levels averaging only 15.6% those observed in noninfected cells) coupled with a reciprocal 72.7% global increase in *Salmonella* tRNA expression levels. In addition to this, these analyses also identified ten sncRNAs (four previously annotated and six unreported) appreciably expressed from the P22 genome during infection (Figure 3, Table 2), and much to our surprise, we found that these bacteriophage-encoded sRNAs actively secreted within host OMVs.

### 3.1. Salmonella sRNAs

We find that 166 sRNAs are appreciably expressed (>25 RPM) in the cytosol of noninfected cells and find that the expression levels of nearly all of these (151 of 166) decreased to less than half their normal levels during P22 infection. Conversely, we find that the cytosolic expression of only one appreciably expressed sRNA, FinP, increased by ≥50% during P22 infection, and strikingly, we find that the expression of this sRNA (known to be directly involved in regulating plasmid transfer [28,29]) is actually increased by over 500% during infection (Table 1). Furthermore, whereas the OMV levels of most *Salmonella* sncRNAs are normally ~1/10 that found in the cytosol [30], we find that P22 infection triggers active OMV encapsulation and secretion of a subset of sRNAs. As specific examples, unlike most sRNAs, the OMV abundances of GcvB and RprA are roughly equal to their cytosolic levels, suggesting the differential loading of and active enrichment in these specific sRNAs within OMVs (Table 1). It is tempting to speculate that P22-driven OMV enrichment in these sRNAs and the subsequent delivery of these to recipients could potentially be involved in facilitating P22 infections in neighboring cells.

### 3.2. Salmonella tRNAs

In stark contrast to sRNAs, P22 infection resulted in an approximate doubling of tRNA abundance in both the cytosol and OMVs (Figure 2, Table 1). The cytosolic expression levels of all tRNAs were increased by at least 50% during P22 infection. That said, while most tRNA OMV abundances were also increased during P22 infection, the expression levels of tRNA^Asp^, tRNA^Met^, and tRNA^Pro^ were markedly depleted in OMVs (Table 1). Interestingly, we found that roughly 50% of RNAs derived from tRNA loci actually correspond to tRNA fragments (tRFs) in cytosolic isolates and roughly 70% in OMV isolates. That said, while we found the overall percentages of reads corresponding to tRNA fragments to be largely unaffected by P22 infection, we did find that the abundances of a number of specific fragments were markedly changed, suggesting that a more comprehensive evaluation focused specifically on tRNA excisions is likely warranted. Similarly to our current study, in 2015, Ghosal et al. found that the majority of RNA contained within *Escherichia coli* OMVs has a length < 60 nt with an enrichment between 15 and 40 nt and that *E. coli* OMV RNA sequencing reads primarily correspond to tRNA fragments (tRFs) [31]. More recently, Koeppen et al. [11] likewise found that tRF levels were significantly higher in RNA isolated from *Pseudomonas aeruginosa* OMVs than in intracellular RNA, suggesting that tRFs are actively enriched in OMVs prior to secretion. Further, of note, despite tRNAs and tRFs typically constituting only ~15% and 2% of normal human intracellular small RNA populations respectively, much like bacterial OMVs, human exosomes have now been reported by several groups as being similarly enriched with tRFs (typically representing >20% of RNA compositions) [32,33,34,35,36]. Despite this, how tRFs are generated and the functional roles of the majority of tRFs remain unclear, although tRFs have been suggested to have initially arisen as a part of an ancient viral defense [37]. Supporting this concept, human endogenous retrovirus transposition rates can be limited by host tRF expression [38], and specific tRFs are induced by viral infections (e.g., by RSV [39,40]). In addition, increasing titers of HIV have been shown to trigger the production of lysine tRFs that inhibit HIV, and T cells infected with human T-cell leukemia virus type 1 (HTLV-1) similarly upregulate the production of proline tRFs targeting this virus [41]. Together, these findings suggest that tRFs may constitute an uncharacterized component of the innate antiviral response. That said, despite there being several published reports now independently describing (1) roles for specific tRFs in facilitating/inhibiting viral replication [39,40,41,42,43], (2) the marked prevalence of tRFs within secreted vesicles (both exosomes and OMVs) [11,33,34,35,36], and (3) the ability of OMVs to inhibit the phage infection of several bacterial species (e.g., *E. coli* [44], *Prochlorococcus marinus* [45], *Shigella flexneri* [46], *Salmonella enterica* [12], and *Vibrio cholerae* [47]), there have been no examinations of the effects of vesicle-secreted tRFs on viral/phage infections reported to date.

### 3.3. P22 sncRNAs

A number of sncRNAs expressed from various phages have been found to regulate specific gene expressions in their respective hosts [48,49,50]. The most notable *Salmonella* P22 sRNAs documented so far include *oop* RNA, a cis-encoded transcriptional repressor whose overexpression likely favors lytic development over lysogenization [17,51], and *sar* asRNA, a trans-encoded transcriptional repressor located in the intergenic region of the ant gene which is also involved in deciding between lytic development or establishing lysogeny [20]. Interestingly, our analyses suggest that at least five of the ten P22 sncRNAs identified in this work are likely involved in regulating the establishment or maintenance of the lysogenic state (Table 3). That said, extensive research detailing the components necessary for successful bacteriophage infections have described many factors contributing to the interactions between bacteriophages and their host bacteria [5]. While the majority of these efforts have been dedicated to exploring protein interactions, our study represents one of the first aimed at comprehensively identifying the phage sncRNAs expressed during these interactions [48,49,50], and to our knowledge, it is the first to ever examine whether phage sncRNAs are actively secreted in bacterial host OMVs. Notably, we find that phage sncRNAs are not simply confined to the cytosol. Instead, our data indicates that all ten of the P22 sncRNAs identified in this work are actively transported into OMVs and secreted into the extracellular space, potentially indicating a co-opting of bacterial OMVs by infecting phages. Notably, the encapsulation of viral sncRNAs and their transfer to neighboring cells to facilitate infection are well documented for a number of human viruses [52,53]. Regardless of whether or not OMV-encapsulated P22 sncRNAs similarly facilitate P22 infections in neighboring cells, the realization that bacteriophage sncRNAs are being secreted in host OMVs during infection reveals a hitherto overlooked dimension of the interactions between bacteriophages and their hosts.

In summary, the work presented here shows that P22 infection triggers a global reduction in *Salmonella* sRNAs coupled with a reciprocal global increase in *Salmonella* tRNA expression levels. Further, we find that P22 infection triggers active OMV encapsulation and secretion of: (1) a subset of *Salmonella* sRNAs, (2) all *Salmonella* tRNAs including one highly complementary to the P22 genome, and (3) ten distinct sncRNAs expressed from P22 itself. That said, while the initial realization that bacteriophage sncRNAs are being loaded into OMVs (and presumably delivered to neighboring cells) is exciting, the elucidation of specific targets and functions for the majority of these sncRNAs remains wholly unresolved and will ultimately require future studies employing experimental methodologies capable of directly characterizing RNA–target interactions.

## 4. Materials and Methods

Strain and growth conditions. *Salmonella* enterica serovar Typhimurium strain LT2 (ATCC 19585) was cultured and maintained on LB agar (1% *w*/*v* tryptone, 1% *w*/*v* NaCl, 0.5% *w*/*v* yeast extract, 1.5% *w*/*v* agar) and incubated overnight at 37 °C. For the preparation of the cell suspension, a loop full bacterial agar culture was inoculated in LB broth (1% *w*/*v* tryptone, 1% *w*/*v* NaCl, 0.5% *w*/*v* yeast extract) and incubated overnight at 37 °C with shaking. For the P22 virus, the lysate was prepared by inoculating 50 µL of *Salmonella* enterica subsp. enterica serovar Typhimurium bacteriophage P22 (ATCC 19585-B1) in a log-phase attained 1:100 diluted overnight culture of *Salmonella* enterica subsp. enterica serovar Typhimurium LT2 (ATCC 19585). Log-phase cultures were used for all infection experiments to ensure efficient and reproducible P22 infection under conditions that support active phage replication. The culture was grown until it became clear, signifying the lysis of host bacterial cells. The culture was filtered and then centrifuged for 10 min at 3000 rpm. The lysate was then transferred into a sterile vial containing 500 µL of chloroform and stored at 4 °C until further use. The PFU/mL of the freshly prepared lysate was counted using a standard Plaque-forming assay protocol.

Infecting *Salmonella* with P22. Log-phase *Salmonella* cultures containing 5.0 × 10^8^ CFU/mL were infected with 100 µL of P22 lysate with a titer of 1.1 × 10^9^ PFU/mL, corresponding to a multiplicity of infection (MOI) of 0.225, then incubated with shaking at 220 rpm for 1 h at 37 °C. Subsequent centrifugation at 5000× *g* for 20 min facilitated the separation of viral particles and cellular components. The resulting supernatant, containing extracellular components, was carefully collected for outer membrane vesicle (OMV) isolation, while the cell suspension containing pellet was stored at −80 °C for later RNA isolation. A control experiment using 50 mL of *Salmonella* log-phase culture without the addition of P22 virus served as baseline.

OMV isolation, visualization, and RNA extraction. OMVs were isolated from the supernatant using the ExoBacteria OMV isolation kit (cat# EXOBAC100A-1, System Biosciences, Palo Alto, CA, USA) in accordance with the manufacturer’s guidelines. RNA was isolated from OMVs and cell pellets using TRIzol (cat# 15596016, Thermo Fisher Scientific, Carlsbad, CA, USA). OMV and cellular RNAs were quantified and assessed using a Qubit 4 Fluorometer (cat# Q33238, Life Technologies Holdings Pte Ltd., Thermo Fisher Scientific, Singapore) and NanoDrop (cat# 840281500,Thermo Fisher Scientific, Waltham, MA, USA) respectively. To confirm the isolation of OMVs, a PMX 220 ZetaView TWIN Laser Nanoparticle tracking analyzer (Particle Metrix GmbH, Bavaria, Germany) was employed to image isolates at 100–200 nm. Samples were diluted 1:10 in ExoBacteria OMV isolation kit elution buffer, and elution buffer alone was run as baseline.

RNA sequencing and analysis. RNA samples isolated on three separate occasions from *Salmonella* cells infected with P22 (C+), OMVs secreted by *Salmonella* infected with P22 (O+), and control samples from noninfected *Salmonella* C− and O− were sent to the High Throughput Sequencing Facility, University of North Carolina, Chapel Hill, where small RNA-seq using an Illumina NextSeq genomic sequencer was performed (single ends, 101 nt, 15M reads). Reads were analyzed using standard Borchert Laboratory protocols [54,55,56,57,58,59,60,61,62]. In brief, raw RNA seq reads were first assessed for quality using FastQC [63]. Adapter sequence (small RNA adapter: 5′-TGGAATTCTCGGGTGCCAAGG-3′), low-quality bases (minimum Phred score 20) were removed using Cutadapt [64], and any reads shorter than 20 nt post-trimming were discarded. FastQC [63] quality analysis was repeated post-trimming to confirm adapter removal and length distributions. The resulting FASTQ sequence files were converted to FASTA format, then reads aligned against the P22 genome (AF217253.1) and LT2 genome (NC_003197.2) with masked ribosomal loci using Standalone BLASTn 2.15.0+ [65]. BLAST parameters were required to be ungapped, have an e value ≤ 0.000001, and have 100% identity across a full alignment of no less than 20 nt. The longest alignment between each read and the genome was identified and all other alignments discarded. Less than 0.02% of reads best mapped to ≥two genomic locations. As each of these multimapping events were found to correspond to specific tRNAs identically represented in the genome, these loci were collectively considered and counted as single alignments. Gene counts were determined by summing the number of aligning reads at each genomic position then averaging the number of aligning reads at each position within Ensembl-defined gene annotations [66].

RT-PCR analyses. RNA samples isolated from *Salmonella* cells infected with P22 (C+), OMVs secreted by *Salmonella* infected with P22 (O+), and control samples from noninfected *Salmonella* C− and O− were used to generate cDNA using Applied Biosystems High-Capacity cDNA Reverse Transcription Kit (cat# 4374967, Thermo Fisher Scientific Baltic UAB, Vilnius, Lithuania). Primer sequences are listed in Supplementary Scheme S2. PCRs were performed with 1 μL (10 μM) forward primer, 1 μL (10 μM) reverse primer, 4 μL MgCl_2_ (25 mM), 1 μL DNTP (10 mM each), 5 μL 10× Taq buffer, 1 μL Taq polymerase (1 U/μL) (cat# FEREP0404, Thermo Fisher Scientific Baltics UAB, Vilnius, Lithuania), and 10 ng cDNA brought to 50 μL with DNA-grade H_2_O (cat# AM9922, Thermo Fisher Scientific, Carlsbad, CA, USA). The thermal cycling parameters for these reactions were an initial denaturation at 95 °C for 1 min, followed by 30 cycles of 30 s at 95 °C, 30 s at 58 °C, and 30 s at 72 °C, then a final extension step at 72 °C for 2 min. PCR products were then run on a 1% agarose TBE gel for 1 h at 90 V and stained with EtBr (cat# BP102-5, Fisher Scientific Company, Fair Lawn, NJ, USA).

## 5. Conclusions

In conclusion, the study presented here shows that P22 bacteriophage infection of its host *Salmonella enterica* causes a reduction in host sRNAs while increasing tRNA abundance simultaneously. In addition, the infection promotes selective encapsulation and presumably, the secretion of distinct RNA molecules via OMV including host sRNAs, tRNAs and ten bacteriophage-derived small non coding RNA (sncRNAs) to the neighboring cells. These findings shed light on the complex nature of OMV mediated communication and warrant further research into the regulatory roles of the secreted RNA species in phage-host dynamics.

## Figures and Tables

**Table 1:** Select *Salmonella* sncRNA expressions. Select housekeeping (top), sRNA (middle), and tRNA (bottom) sncRNAs. sncRNA, name based on Ensembl annotation except for “tRNA^thr^-attP”, which is identically represented in both the *Salmonella* and P22 genomes. RPM, reads per million. C−, RNA reads isolated from *Salmonella* cells not infected with P22; C+, RNA reads isolated from *Salmonella* cells infected with P22; O−, RNA reads isolated from OMVs secreted from *Salmonella* cells that were not infected with P22; O+, RNA reads isolated from OMVs secreted from *Salmonella* cells infected with P22. *p* values of two-sample *t*-tests comparing C+/C− and O+/O− are indicated. “ns”, not significant (*p* value ≥ 0.05).

sncRNA	RPM				*p* Value	
	C−	C+	O−	O+	C+/C−	O+/O−
Rnase P	63	50	118	77	ns	ns
srpRNA	115	439	29	153	ns	ns
tmRNA	362	394	272	254	ns	ns
ArcZ	893	93	43	12	0.0303	ns
CopA	5596	423	63	12	ns	0.0017
CsrC	240	42	19	13	ns	ns
DapZ	1639	63	10	3	0.0214	ns
FinP	51	256	25	43	0.0406	ns
FnrS	377	17	3	5	0.0195	ns
GcvB	221	24	46	15	0.0447	0.0001
InvR	258	68	15	8	0.0127	ns
IsrB2	29	8	30	1	ns	0.0286
MgrR	316	40	47	9	0.0398	0.0154
MicA	528	65	16	1	ns	ns
npcRNA44	825	86	306	30	ns	0.0126
RprA	121	41	23	23	ns	ns
RybB	152	17	18	1	0.0415	0.0001
RyeC	228	59	172	6	ns	0.0002
sroB	1063	136	52	14	0.0432	0.0005
SsrS	5276	1926	213	520	ns	ns
tRNA^His^	2825	4860	1503	3559	ns	ns
tRNA^Thr^-attP	7068	10,362	2518	7895	ns	ns
tRNA^Phe^	9814	14,894	1870	8838	ns	ns
tRNA^Lys^	1705	5276	976	1399	ns	ns
tRNA^Try^	1105	2188	298	643	ns	ns
tRNA^Asp^	873	1708	2798	534	0.0003	0.0117
tRNA^Met^	736	1166	808	429	ns	0.0403
tRNA^Gly^	8061	15,988	3395	10,834	0.0120	ns
tRNA^Thr^	3377	4572	1652	3219	ns	ns
tRNA^Pro^	153	450	1480	854	0.0055	ns
tRNA^Sec^	47	319	22	133	ns	ns

**Table 2:** SncRNAs expressed from P22 genome. sncRNA, arbitrary name based on position in P22 genome. If applicable, names of previously reported sncRNAs are included following arbitrary designation assigned by this study. P22 nt Start, start position of RNA in P22 genome. P22 nt End, end position of RNA in P22 genome; P22 strand, strand (− or +) that sncRNA is expressed from; RNA Sequence, sequence that corresponds to denoted start and stop positions in P22 genome. Sal (Y/N) indicates whether or not sequence is also present in *Salmonella* genome. RPM, reads per million. O−, RNA reads isolated from OMVs secreted from *Salmonella* cells that were not infected with P22; C−, RNA reads isolated from *Salmonella* cells not infected with P22; O+, RNA reads isolated from OMVs secreted from *Salmonella* cells infected with P22; C+, RNA reads isolated from *Salmonella* cells infected with P22.

sncRNA	P22 nt Start	P22 nt End	Length	P22 Strand	RNA Sequence	Sal (Y/N)	RPM			
							O−	C−	O+	C+
P22A-sas	11,387	11,513	127	-	UUCAUCGGCGAUUCUCUUUUUACUCUCUGUAGGGGUGAAUAGAGUUUAUCCGAUUUCUCGCUGUAGGGGUACACGAGAACCACCGAGCCUGACGUGGUUAAAAGACAGGCACAAUCUUUACUACCGC	N	0	0	139	1321
P22B	12,426	12,521	96	-	CUUAGGCCUCUAGCUGUACCGAUCGGGCCGGACUGAGAAGCCACUUGAAAUCCGGAAAUUGAGACAGGUUCCGGCGCCAGUACCAAAGCCAUUUCA	N	0	0	216	642
P22C-oop	14,024	14,086	63	-	GAUUGAUCCAGUCUUUCUACAUCAGGCCUCAAAACUGUUCCCGCAGUCUUGAGGCUUUUCUUU	N	0	0	653	1513
P22D-23as	19,082	19,125	44	-	UUUGUCGUUCUGGCUGAGUUCGUGCUUACCGCAGAAUGCAGCCA	N	0	0	17	132
P22E	19,716	19,816	101	+	UUCUGAAAGCGCCCUAUCACCAAUCACCAGAACACAUCCAGAUACCCUUGCUCAUUCGUGGCGACGGGGUAGGGCGUUUUACACAAAAGAAAACCCAGAAC	N	0	0	1883	2582
P22F	21,301	21,389	89	+	AAUGGGCCUUUGAGGAUACCAGUUAGUGCUGGCGAGCCUCGGUGGGCUGGUUUCCUGUGCGGCAAAGGUUCAUUUCAAAGAAGAAGGCA	N	0	0	926	2054
P22G	21,873	22,076	204	+	CGAGAAUUGGACGCCUUGAACAGCACGGGCAAACCGUAAUCCCCGGGCUCACUAAUUAACGGCAGGACAGCGAAACAACCCAAGCCAGUAAGUGGGGGAAAAUAACACUGGCAGCCACUGAAAGAUGAACCUCCAGCCGUAUGGCAAAAAAGAUUCUUUGUGGUGGCGGACUGAUGGAAAGACAUCGGUUAUUGCAGAGGCCAU	N	0	0	374	488
P22H	27,230	27,351	122	+	CAAAUGCUGAGUUACUCCUUAAAGGCGAUGAACAGACGCACAAGCAGCGAAUGGACAUUGCCAACAUCCUGCAAUCGCAGAGACAAAAUCAACCUUCCGGCAGUGUAGCCGAGACACCUCAA		0	0	267	166
P22I	28,204	28,256	53	+	AAUGGAUGCUGCUGCGAGCAAGGGAGAUGUGGAAACCUACCGCAAGCUAAAGG	N	0	0	847	400
P22J-sar	38,468	38,571	104	-	AUUCAUGUUGGUUUUUCUCCAAGGAUUUACUGACAACCGAAGCCCUGACUGUUACCGCAGUUGGGGCUUCAACUUUACGCGCCAAUGCGCCCUUCCUUCUUAAA	N	0	0	1433	2724

**Table 3:** P22 sncRNA putative targets. sncRNA, arbitrary name based on the position in the P22 genome. Putative target/function, presumed targets based on being either antisense to or just upstream of a P22 protein-coding gene. The known functions of characterized P22 sncRNAs and/or functions of homologous protein targets in other bacteriophages are included if available.

sncRNA	Putative Target/Function
P22A-sas	Previously characterized antisense regulator of SieB. *sas* asRNA induces a translational switch between distinct peptides encoded by the sieB gene critical to SieB-mediated superinfection exclusion [16].
P22B	16 bp upstream of Gene 24. Gene 24 is an early protein lambda N homolog that functions as a transcriptional antiterminator [2]. The expression of most lambda early genes depends on the formation of a N protein-dependent antiterminating transcription complex [23].
P22C-oop	Previously characterized antisense regulator of C1. The transcription activator protein C1 plays a key role in the lytic versus lysogenic switch of the phage [17,18].
P22D-23as	Previously characterized antisense regulator of Gene 23 (analogous to lambda Q). P22 utilizes antisense regulation to turn down Gene 23 expression in a C1-dependent manner in support of lysogenic growth [19].
P22E	48 bp upstream of Gene 13. Gene 13 of bacteriophage P22 is functionally equivalent to lambda lysis gene S holin, and the suppression of Gene 13 is likely involved in the establishment or maintenance of the lysogenic state [24,25].
P22F	Partially overlapping ORF201 [2]. No known function.
P22G	Downstream of ORF201 [2]. No known function.
P22H	17 bp upstream of Gene 1 (portal protein). The Gene 1 protein of *Salmonella* bacteriophage P22 is located at the DNA packaging vertex of the mature particle. The protein is incorporated into the procapsid shell during shell assembly and is required for DNA packaging [26].
P22I	23 bp upstream of Gene 5 (coat/capsid protein). The assembly of the procapsid shell of phage P22 requires the Gene 5 coat protein [27].
P22J-sar	Previously characterized antisense regulator that negatively regulates the production of the antirepressor protein (Ant) by basepairing with its mRNA, preventing its translation. The balance of antirepressor and repressor activity influences whether the phage enters the lytic cycle (rapid production of progeny) or establishes lysogeny [20].

## Data Availability

All small RNA-sequencing datasets generated were deposited in the NCBI SRA repository. Two sets of replicates are available under Bioproject PRJNA1307193 (SRR35017900, SRR35017901, SRR35017902, SRR35017903, SRR35996074, SRR35996075, SRR35996076, SRR35996077), and a third set of replicates is available under Bioproject PRJNA1141709 (SRR30031405, SRR30031404, SRR30031403, SRR30031402). All other relevant data (e.g., alignment files) are available from the authors upon request.

## Supplementary Materials

The following supporting information can be downloaded at https://www.mdpi.com/article/10.3390/ncrna12040021/s1, Supplemental Table S1: P22 sncRNAs identically maintained in other bacteriophage genomes. sncRNA, arbitrary name based on position in P22 genome. “Bacteriophage genomes containing the full length, identical sequence” lists bacteriophage genomes bearing 100% identity to indicated P22 RNAs as determined using NCBI Virus [67]. IDs correspond to phage published in NCBI Reference Sequence Database (RefSeq) [68]. Individual phage IDs are separated by commas, and primary host specificity is indicated and separated by semicolons. Supplemental Scheme S1: ZetaView of OMV isolates. (A) OMV from O− sample (left) and from O+ sample (right). (B) OMV from O− sample (left) and from O+ sample (right) following Proteinase K (PK) treatment. (C) Buffer control (left) and 125 Thermo Scientific 3000 Series Nanosphere Size Standard (right). Supplemental Scheme S2: RT-PCR validation of P22 sncRNAs. RT-PCR performed on RNA isolates from P22-infected (C+) and -noninfected (C-) *Salmonella*. (Top left) Sequences of putative P22 sncRNAs identified by sequence read alignments are shown to right of their genomic position and strand. (Bottom left) Putative P22 sncRNA sequences are highlighted within their local genomic sequences along with color-coded primers employed for RT-PCR. F and R primers were designed within called sncRNA. F_out_ and R_out_ primers were designed in sequences flanking called sncRNA. (Right) PCR amplicons visualized after separation on 1.0% agarose gels stained with EtBr. (A) P22F. (B) P22H.
