# Supplementary material for: P22 Small Noncoding RNAs Are Actively Secreted in Salmonella Outer Membrane Vesicles During Bacteriophage Infection
Source: Noncoding RNA. 2026 Jun 26;12(4):21. doi: 10.3390/ncrna12040021 (PMC13397905; doi:10.3390/ncrna12040021)
Supplement: Supplementary file 1 [file ncrna-12-00021-s001.zip › ncrna-4282361-supplementary.pdf]

Supplemental Table S1. P22 sncRNAs identically maintained in other bacteriophage genomes. sncRNA, arbitrary name based on position in P22 genome. "Bacteriophage genomes containing the full length, identical sequence" lists bacteriophage genomes bearing 100% identity to indicated P22 sncRNAs as determined using NCBI virus [67]. IDs correspond to phage published in NCBI Reference Sequence Database (RefSeq) [68]. Individual phage IDs are separated by commas, and primary host specificity is indicated and separated by semicolons.

sncRNA	Bacteriophage genomes containing the full length, identical sequence
P22A-sas	*Salmonella* phages: 25, 34, AS1, AS3, SE10, SE13, SE21, SF3, SF11, SI23, ST35, vB_SemP_ER25, VSe13; other enterobacteriaceae phages: HT105/1 int-201, Phi75, UAB_Phi20
P22B	*Salmonella* phages: 25, 34, As1, As3, HT105/1 int-201, SE10, SE16, SE21, SE22, SF11, SF3, SI23, ST-35, vB_SemP_Emek, vB_SenP_ER25, VSe13; other enterobacteriaceae phages: ES18, Phi75, UAB_Phi20
P22C-oop	*Salmonella* phages: 25, 34, As1, As3, HT105/1 int-201, MG40, SE10, SE16, SE21, SE22, SF11, SF3, SI23, ST-35, vB_SenP_ER25, VSe13; other enterobacteriaceae phages: Phi75, UAB_Phi20
P22D-23as	*Salmonella* phages: 25, 34, As1, As3, BIS08P22, HT105/1 int-201, vB_Se_GLBO_sp11, vB_SenP_ER25, VSe13; other enterobacteriaceae phages: O276, 4W, DE3, DN1, E26-Berryhill, HK106, HK544, HK630, Lambda, Lambda h434 imm21, Lambda imm21, Lambda imm434, Phi75, UAB_Phi20; Caudoviricetes phage; *Campylobacteraceae* phage CJLB-4
P22E	*Salmonella* phages: As1, As3, HT105/1 int-201, vB_Se_GLBO_sp11, vB_SenP_ER25, VSe13; other enterobacteriaceae phages: Phi75, UAB_Phi20
P22F	*Salmonella* phages: 25, 34, As1, As3, HT105/1 int-201, vB_SenP_ER25; other enterobacteriaceae phages: Phi75, UAB_Phi20
P22G	*Salmonella* phages: 25, 34, As1, As3, HT105/1 int-201, vB_SenP_ER25; other enterobacteriaceae phages: Phi75, UAB_Phi20
P22H	*Salmonella* phages: 101962B_sal5, 103203_sal4, 118970_sal4, 146851_sal4, 25, 34, 64795_sal4, As1, As3, BIS08P22, HT105/1 int-201, S.Kent 1-2-1, SEN35, SF10, SF6, Si3 KFo-2019, SI5, SI8, SPLA1b, SPN9CC, ST160, ST-29, ST-32, ST-35, UPF_BP1, vB_Se_GLBO_sp11, vB_SemP_Emek, vB_SenP_ER25, VSe13; other enterobacteriaceae phages: Phi75, UAB_Phi20
P22I	*Salmonella* phages: 101962B_sal5, 103203_sal4, 118970_sal4, 146851_sal4, 25, 34, 64795_sal4, As1, As3, HT105/1 int-201, S.Kent 1-2-1, S9-5, UPF_BP1, vB_SalP_ABTNLsp11242, vB_Se_GLBO_sp11, vB_SenP_ER25, VSe13; *Escherichia* phage ECO95 1-6-1
P22J-sar	*Salmonella* phages: 101962B_sal5, 103203_sal4, 118970_sal4, 146851_sal4, 25, 64795_sal4, As1, As3, HT105/1 int-201, SE10, SE16, SE21, SE22, SF11, SF3, SI23, vB_SenP_ER25; other enterobacteriaceae phages: Phi75, UAB_Phi20

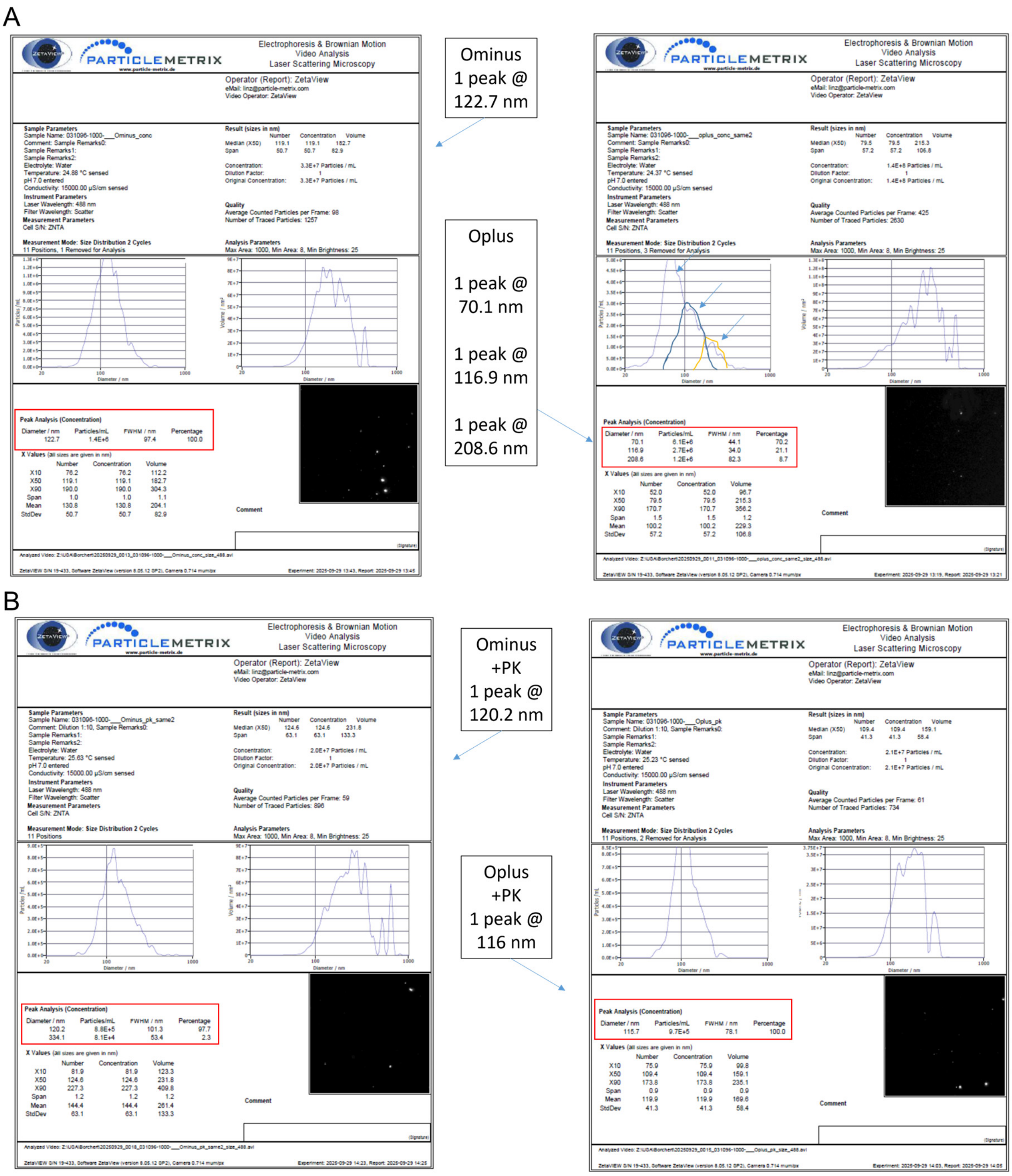
A
Ominus
1 peak @
122.7 nm
Oplus
1 peak @
70.1 nm
1 peak @
116.9 nm
1 peak @
208.6 nm
B
Ominus
+PK
1 peak @
120.2 nm
Oplus
+PK
1 peak @
116 nm

C

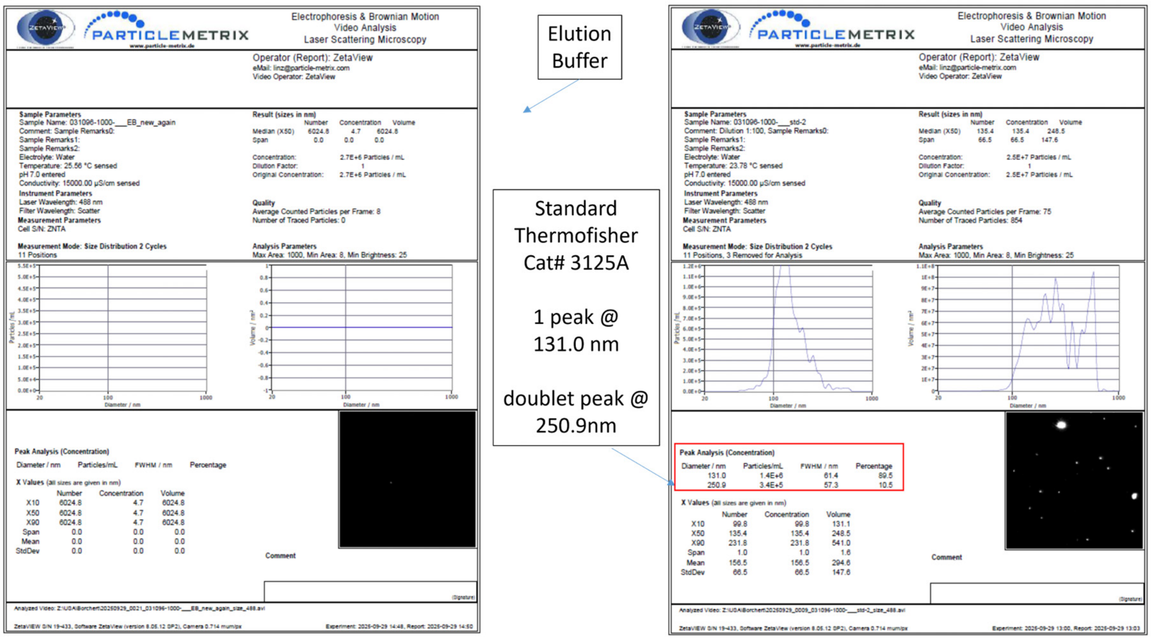

Scheme S1. ZetaView of OMV isolates. (**A**) OMV from O- sample (left) and from O+ sample (right). (**B**) OMV from O- sample (left) and from O+ sample (right) following Proteinase K (PK) treatment. (**C**). Buffer control (left) and 125 Thermo Scientific 3000 Series Nanosphere Size Standard (right).

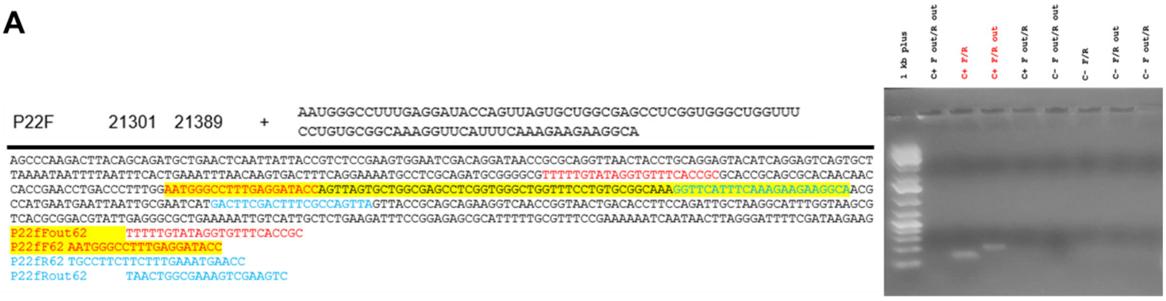

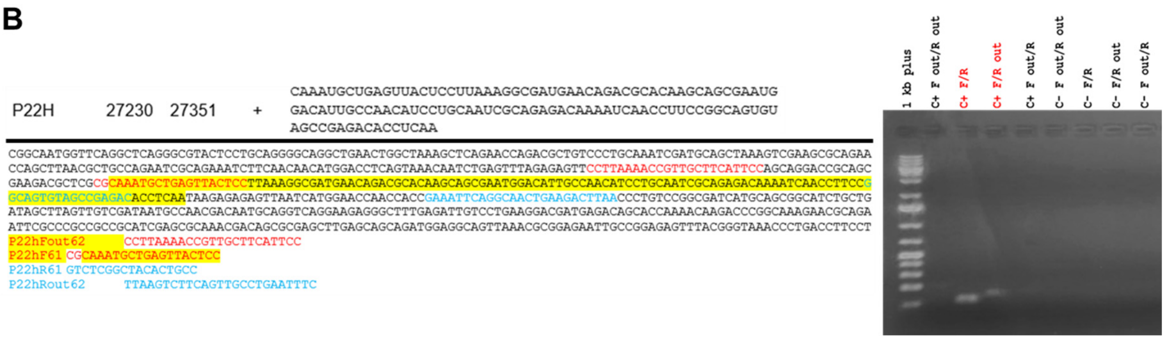

Scheme S2. RT-PCR validation of P22 sncRNAs. RT-PCR performed on RNA isolates from P22-infected (C+) and -noninfected (C-) *Salmonella*. (Top left) Sequences of putative P22 sncRNAs identified by sequence read alignments are shown to right of their genomic position and strand. (Bottom left) Putative P22 sncRNA sequences are highlighted within their local genomic sequences along with color-coded primers employed for RT-PCR. F and R primers were designed within called sncRNA. F_{out} and R_{out} primers were designed in sequences flanking called sncRNA. (Right) PCR amplicons visualized after separation on 1.0% agarose gels stained with EtBr. (**A**) P22F. (**B**) P22H.
